# Nitrate Reductases Are Relocalized to the Nucleus by AtSIZ1 and Their Levels Are Negatively Regulated by COP1 and Ammonium

**DOI:** 10.3390/ijms19041202

**Published:** 2018-04-15

**Authors:** Joo Yong Kim, Bong Soo Park, Sang Woo Park, Han Yong Lee, Jong Tae Song, Hak Soo Seo

**Affiliations:** 1 Department of Plant Science, Research Institute of Agriculture and Life Sciences, Seoul National University, Seoul 151-921, Korea; kjy90kjy@snu.ac.kr (J.Y.K.); bongsoo@tll.org.sg (B.S.P.); nn4878@snu.ac.kr (S.W.P.); 2 Department of Botany and Plant Pathology, Purdue University, West Lafayette, IN 47907, USA; lee2224@purdue.edu; 3 School of Applied Biosciences, Kyungpook National University, Daegu 41566, Korea; jtsong68@knu.ac.kr; 4 Plant Genomics and Breeding Institute, Seoul National University, Seoul 151-921, Korea; 5 Bio-MAX Institute, Seoul National University, Seoul 151-818, Korea; 6 Department of Plant Science, College of Agriculture and Life Sciences, Seoul National University, Gwanak-ro 1, Gwanak-gu, Seoul 151-921, Korea

**Keywords:** nitrate reductase, AtSIZ1, nitrate, ammonium, localization, NIA1, NIA2, COP1

## Abstract

Nitrate reductases (NRs) catalyze the first step in the reduction of nitrate to ammonium. NR activity is regulated by sumoylation through the E3 ligase activity of AtSIZ1. However, it is not clear how NRs interact with AtSIZ1 in the cell, or how nitrogen sources affect NR levels and their cellular localization. Here, we show that the subcellular localization of NRs is modulated by the E3 SUMO (Small ubiquitin-related modifier) ligase AtSIZ1 and that NR protein levels are regulated by nitrogen sources. Transient expression analysis of GFP fusion proteins in onion epidermal cells showed that the NRs NIA1 and NIA2 localize to the cytoplasmic membrane, and that AtSIZ1 localizes to the nucleoplasm, including nuclear bodies, when expressed separately, whereas NRs and AtSIZ1 localize to the nucleus when co-expressed. Nitrate did not affect the subcellular localization of the NRs, but it caused AtSIZ1 to move from the nucleus to the cytoplasm. NRs were not detected in ammonium-treated cells, whereas the localization of AtSIZ1 was not altered by ammonium treatment. NR protein levels increased in response to nitrate but decreased in response to ammonium. In addition, NR protein levels increased in response to a 26S proteasome inhibitor and in *cop1-4* and DN-COP1-overexpressing transgenic plants. NR protein degradation occurred later in *cop1-4* than in the wild-type, although the NR proteins did not interact with COP1. Therefore, AtSIZ1 controls nuclear localization of NR proteins, and ammonium negatively regulates their levels. The function and stability of NR proteins might be post-translationally modulated by ubiquitination.

## 1. Introduction

Nitrogen is essential for plant growth and development, as both an inorganic element and a component of secondary metabolites, proteins, and nucleic acids. Plants take up nitrate via nitrate transporters, and reduce it to nitrite through nitrate reductases (NRs). These enzymes function as homodimers in an NAD(P)H-dependent manner. Nitrite is further reduced to ammonium, a component of amino acids [1,2,3]. The *Arabidopsis thaliana* genome contains two NR genes, *NIA1* and *NIA2*, which encode NR 1 and 2, respectively [4].

AtSIZ1 is an Arabidopsis E3 SUMO ligase with self-sumoylation and substrate sumoylation activity. AtSIZ1 contains an SP (Siz/PIAS)-RING (Really Interesting New Gene) finger motif, a SAP (SAF-A/B, Acinus, and PIAS) domain, and a Miz (Msx-interacting-zinc finger) domain [5]. SUMO is a small ubiquitin-related modifier that covalently attaches to its target lysine. Modification of target proteins by SUMO can be directly performed by the SUMO-conjugating enzyme E2 [6]; however, in many cases, E3 SUMO ligase catalyzes the modification of target proteins by SUMO [7]. In Arabidopsis, many SUMO conjugates have been identified by proteomics and yeast two-hybrid screening [8,9]. However, only a few proteins, including AtSIZ1 [10], AtSCE1, Arabidopsis SUMO conjugating E2 enzyme [10], FLOWERING LOCUS C (FLC) [11,12], CHROMOMETHYLASE 3 (CMT3) [13], and SLEEPY 1 (SLY1) [14] are proven experimentally to be sumoylated. AtSIZ1 is involved in germination [15], phosphate responses [16], nitrogen assimilation [17], gibberellin signaling [14], flowering [11,12,18], abiotic stress responses [19,20,21,22,23,24,25], DNA methylation [13], and cell growth and development [26].

NR is a major controller of nitrogen assimilation in plants; however, few factors and/or regulatory mechanisms that influence NR activity have been identified. Arabidopsis NR is inactivated rapidly by phosphorylation [27,28,29], and degradation of spinach NR is regulated by 14-3-3 proteins [30]. Overexpression of the Arabidopsis SNF1-related kinase SnRK1.1 increases the amount of phosphorylated NR, an inactive form of NR [31]. Arabidopsis nitrate reductase NIA1 and NIA2 are modified by SUMO through the E3 SUMO ligase activity of AtSIZ1, and sumoylation increases the nitrate reduction activity of NIA1 and NIA2 [17].

NIA1 and NIA2 are cytoplasmic proteins [32], and AtSIZ1 is a nuclear protein [16,24]. In addition, most SUMO conjugates that require AtSIZ1 are found in the nucleus [33]. Nevertheless, both NIA1 and NIA2 are sumoylated by AtSIZ1 through direct interaction [17], strongly suggesting that AtSIZ1 and NR proteins interact in a specific subcellular organ under specific conditions. 

Expression of *NIA1* and *NIA2* is induced by nitrate [34]. Chromomethylase 3 (CMT3)-mediated methylation of *NIA2* is inhibited by ammonium, resulting in upregulation of *NIA2* expression, whereas the methylation level of *NIA1* is not affected by ammonium treatment [35]. NR genes are highly expressed in *cop1* [36], and expression of *NIA2* is positively controlled by LONG HYPOCOTYL 5 (HY5) and HY5 HOMOLOG (HYH) [37]. COP1 (CONSTITUTIVE PHOTOMORPHOGENIC 1) is a RING finger type E3 ubiquitin ligase that has self-ubiquitination and substrate ubiquitination activity for various targets [38]. Dominant-negative COP1 (DN-COP1)-overexpressing Arabidopsis mimics the phenotypes of *cop1* mutant and accumulates photoreceptor phytochrome A as in *cop1* mutants [39]. However, it is not clear how NR protein levels are regulated at the translational and post-translational levels. The effects of COP1, nitrate, and ammonium on NR protein levels also remain unknown.

Here, we investigated the effect of AtSIZ1 on subcellular localization of NIA1 and NIA2, and the effects of exogenous nitrogen sources and COP1 on the levels of these proteins. We found that NIA1 and NIA2 are relocalized to the nucleus by AtSIZ1 and that their levels are upregulated by nitrate but downregulated by ammonium. We also found that NR protein levels are negatively regulated by COP1, although in an indirect manner. 

## 2. Results

### 2.1. NR Proteins and AtSIZ1 Localize to the Nucleus

Previously, we found that NIA1 and NIA2 interact directly with AtSIZ1, and are sumoylated by its E3 SUMO ligase activity, resulting in marked increases in NR activity [17]. This finding suggests that NR proteins and AtSIZ1 are localized to the same subcellular compartment. Localization studies with GFP fusion proteins show that most, if not all, of the AtSIZ1 in a cell localizes to the nucleus in the form of speckles [16,24]. NIA1 and NIA2 localize to the cytoplasm and plasma membrane [32,40,41]. In addition, most SUMO1/2 conjugates that require AtSIZ1 are localized to the nucleus [33]. 

**Figure 1:**
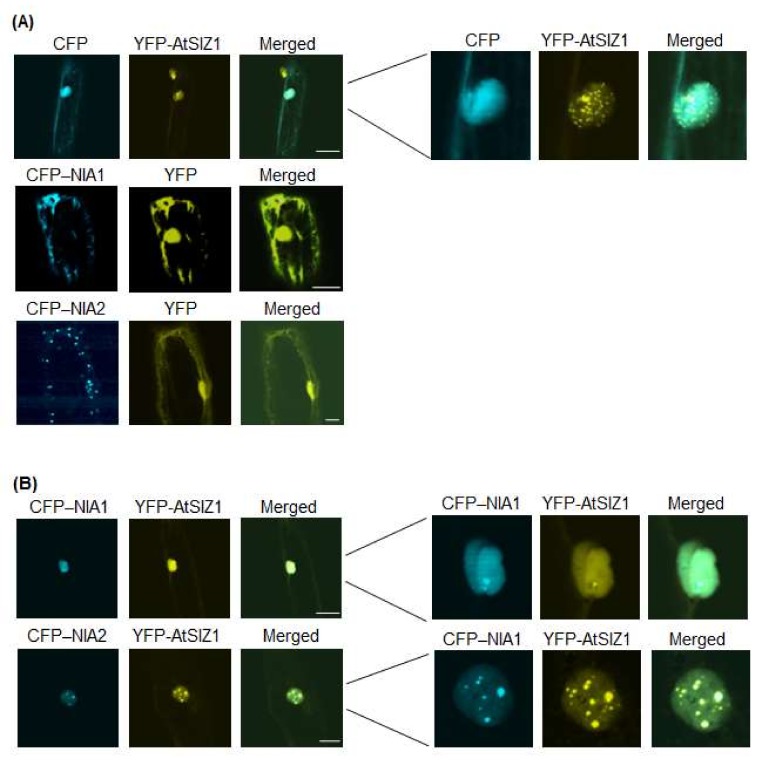
Subcellular localization of AtSIZ1 and NR proteins NIA1 and NIA2. Localization of NIA1, NIA2, and AtSIZ1 was investigated by particle bombardment of onion epidermal cells. Constructs harboring *35S-CFP-NIA1*, *35S-CFP-NIA2*, and *35S-YFP-AtSIZ1* were introduced into onion epidermal cells by particle bombardment alone (**A**) or in combination (**B**). After incubation at 25 °C in the dark for 16 h, the bombarded tissues were analyzed by confocal microscopy to visualize transient CRP or YFP expression. CFP-NIA1, CFP-NIA2, and YFP-AtSIZ1 refer to CFP and YFP fused to the C-terminus of NIA1, NIA2, and AtSIZ1, respectively. DIC, differential interference contrast. Bar, 50 μm.

Therefore, in the current study, we examined the localization of AtSIZ1, NIA1, and NIA2 by particle bombardment of onion epidermal cells. When onion cells were bombarded with cyan fluorescent protein (CFP)-NIA1 or CFP-NIA2 individually, both CFP-NIA1 and CFP-NIA2 localized to the cytoplasmic membrane (Figure 1A). Yellow fluorescent protein (YFP)-AtSIZ1 localized to the nucleus, including nuclear bodies and nucleoplasm (Figure 1A). However, upon co-bombardment with YFP-AtSIZ1 and CFP-NIA1 or CFP-NIA2, both CFP-NIA1 and CFP-NIA2 moved to the nucleus and localized to the nucleoplasm (Figure 1B). In addition, NIA1 and NIA2 also appeared to form speckles (Figure 1B). AtSIZ1 localized to the nucleus, including nuclear bodies (Figure 1B). 

### 2.2. Nuclear Localization of NR Proteins Is Not Affected by Nitrate

**Figure 2:**
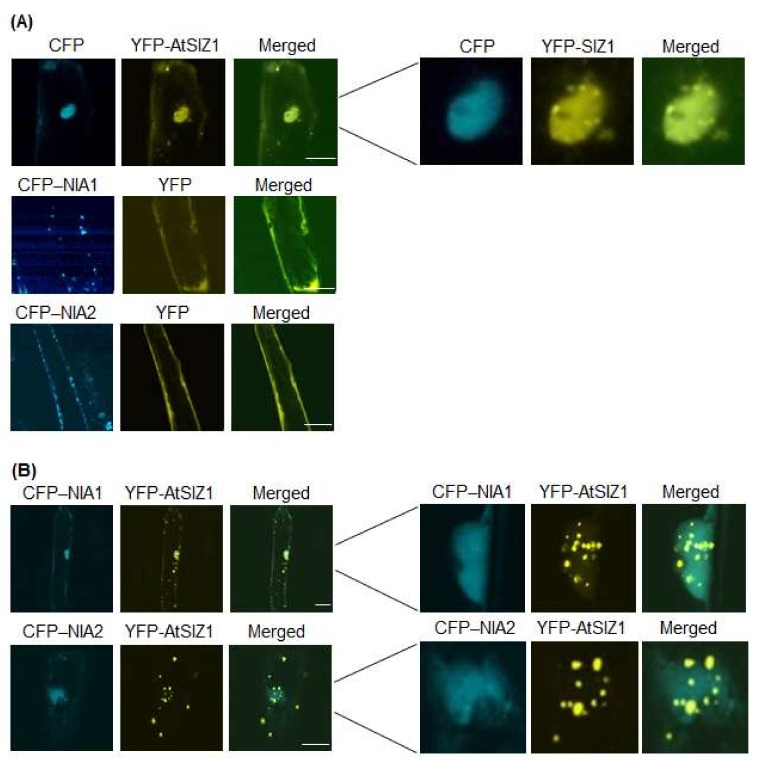
Effect of nitrate on subcellular localization of AtSIZ1 and NR proteins NIA1 and NIA2. Localization of NIA1, NIA2, and AtSIZ1 was investigated by particle bombardment of onion epidermal cells. Constructs harboring *35S-CFP-NIA1*, *35S-CFP-NIA2*, and *35S-YFP-AtSIZ1* were introduced into onion epidermal cells, either alone (**A**) or in combination (**B**), by particle bombardment. After incubation at 25 °C in the dark for 16 h in 5 mM KNO_3_, the bombarded tissues were analyzed by confocal microscopy to visualize transient CRP or YFP expression. CFP-NIA1, CFP-NIA2, and YFP-AtSIZ1 refer to CFP and YFP fused to the C-terminus of NIA1, NIA2, and AtSIZ1, respectively. DIC, differential interference contrast. Bar, 50 μm.

Plants acquire nitrate from the soil, which is trafficked across membranes by nitrate transporters and reduced to nitrite by nitrate reductases, suggesting that nitrate levels influence the localization or levels of NR proteins. Therefore, we examined the effect of nitrate on localization of NIA1 and NIA2. Specifically, we bombarded onion epidermal cells with constructs harboring *35S-CFP-NIA1*, *35S-CFP-NIA2*, and *35S-YFP-AtSIZ1,* either alone or in combination, and treated them with 5 mM KNO_3_. CFP-NIA1 or CFP-NIA2 localized to the cytoplasmic membranes when bombarded individually (Figure 2A), whereas YFP-AtSIZ1 localized to the nucleus, including nuclear bodies (Figure 2A). However, when the cells were treated with nitrate solution after co-bombardment with YFP-AtSIZ1 and CFP-NIA1 or CFP-NIA2, both CFP-NIA1 and CFP-NIA2 were distributed in the nucleoplasm (Figure 2B). Interestingly, localization of YFP-AtSIZ1 was altered by nitrate treatment. Some YFP-AtSIZ1 fluorescent signals were still found in the nucleus as nuclear bodies, and some YFP-AtSIZ1 signals were also found in the cytoplasm as cytoplasmic bodies (Figure 2B). These results suggest that nitrate relocalizes AtSIZ1 from the nucleus to the cytoplasm to allow its E3 ligase activity to be used against other substrates. 

### 2.3. NIA1 and NIA2 Are Not Detected in Ammonium-Treated Cells

**Figure 3:**
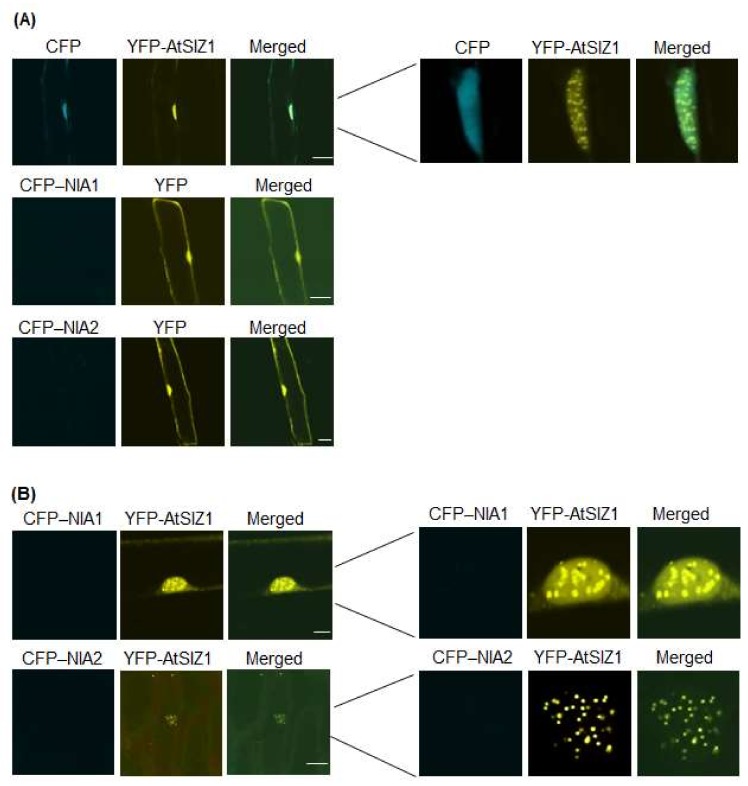
Effect of ammonium on subcellular localization of AtSIZ1 and NR proteins NIA1 and NIA2. Localization of NIA1, NIA2, and AtSIZ1 was investigated by particle bombardment of onion epidermal cells. Constructs harboring *35S-CFP-NIA1*, *35S-CFP-NIA2*, and *35S-YFP-AtSIZ1* were introduced into onion epidermal cells, either alone (**A**) or in combination (**B**), by particle bombardment. After incubation at 25 °C in the dark for 16 h in 5 mM (NH_4_)_2_SO_4_, bombarded tissues were analyzed by confocal microscopy to visualize transient expression. CFP-NIA1, CFP-NIA2, and YFP-AtSIZ1 refer to CFP and YFP fused to the C-terminus of NIA1, NIA2, and AtSIZ1, respectively. DIC, differential interference contrast. Bar, 50 μm.

Ammonium is the end product produced from nitrate via NR activity, and it enters the amino-acid pool primarily via the action of glutamine synthetase. Therefore, ammonium can control the level and localization of NR proteins. Therefore, we investigated the effect of ammonium on localization of NIA1 and NIA2. We bombarded onion epidermal cells with constructs harboring *35S-CFP-NIA1*, *35S-CFP-NIA2*, and *35S-YFP-AtSIZ1,* either alone or in combination, and treated the cells with 5 mM (NH_4_)_2_SO_4_. Fluorescence generated by CFP-NIA1 and CFP-NIA2 was undetectable when bombarded into cells individually (Figure 3A), whereas YFP-AtSIZ1 still localized to the nucleus, including nuclear bodies (Figure 3A). In addition, neither CFP-NIA1 nor CFP-NIA2 signals were detected, even when co-bombarded into onion cells (Figure 3B). However, YFP-AtSIZ1 was clearly observed in the nucleus, including nuclear bodies, when co-bombarded with the other constructs (Figure 3B). 

### 2.4. NIA1 and NIA2 Levels Are Regulated by Nitrate and Ammonium Sources

Nitrate is a substrate of NR proteins, and ammonium is an end product generated by NR activity. Nitrate induces expression of *NIA1* and *NIA2* [34]. In addition, we found that ammonium treatment caused a marked reduction in CFP-NIA1 and CFP-NIA2 signals (Figure 3). These findings prompted us to investigate the effects of nitrate and ammonium on expression of *NIA1* and *NIA2*. First, we examined their effects on expression of *NIA1* and *NIA2* at the transcriptional level. We examined *NIA1* and *NIA2* transcript levels by quantitative real-time polymerase chain reaction (qRT-PCR) using total RNA isolated from the leaves and roots of wild-type plants treated with 5 mM KNO_3_ or (NH_4_)_2_SO_4_. Levels of *NIA1* and *NIA2* transcripts increased in KNO_3_- or (NH_4_)_2_SO_4_-treated leaves and roots (Table 1), but were unchanged in K_2_SO_4_-treated leaves and roots, which served as controls. 

**Figure 4:**
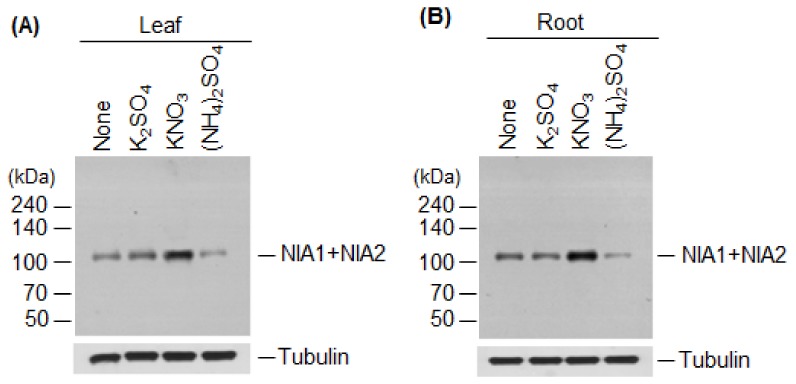
Effect of nitrate or ammonium ion on nitrate reductase levels. The effect of nitrate or ammonium sources on NIA1 and NIA2 protein levels was examined. Wild-type plants were treated with 5 mM K_2_SO_4_, KNO_3_, or (NH_4_)_2_SO_4_, and total proteins extracted from leaves (**A**) or roots (**B**). NR protein (NIA1 + NIA2) levels were examined by immunoblotting with an anti-NR antibody. Tubulin was used as a loading control.

We then examined the effects of nitrate and ammonium on the levels of NR proteins at the translational level. Specifically, we performed immunoblot analysis to measure NR protein levels using total proteins extracted from the leaves and roots of wild-type plants treated with 5 mM K_2_SO_4_, KNO_3_, or (NH_4_)_2_SO_4_. Compared with the control, NR protein levels were higher in KNO_3_-treated leaves and roots (Figure 4A,B) but, interestingly, they were significantly reduced in (NH_4_)_2_SO_4_-treated leaves and roots (Figure 4A,B). NR protein levels were unchanged in K_2_SO_4_-treated control leaves and roots. 

### 2.5. NR Proteins Accumulate in cop1-4 and DN-COP1-Overexpressing Plants

**Figure 5:**
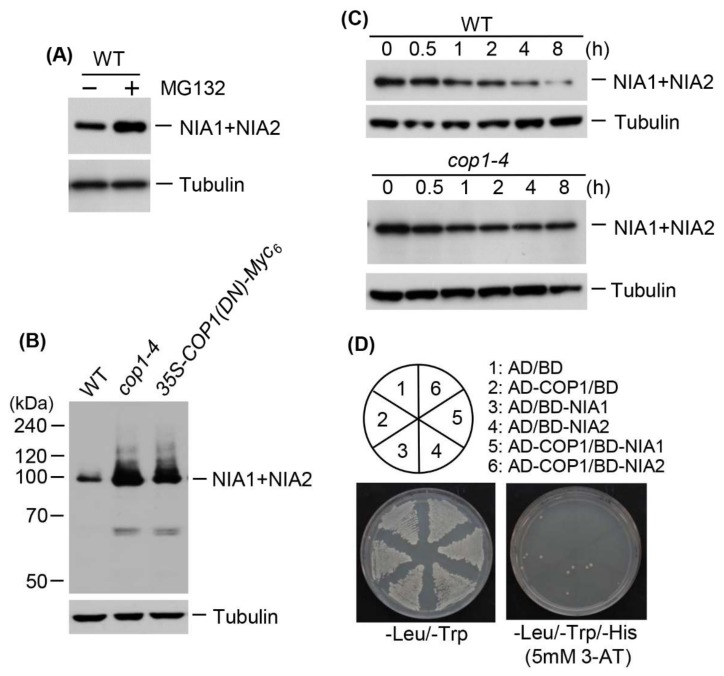
Effect of COP1 on nitrate reductase levels. (**A**) Total proteins were extracted from wild-type plants treated with 100 μM MG132, and NR proteins (NIA1 + NIA2) were detected by immunoblotting with an anti-NR antibody; tubulin was used as a loading control; (**B**) Examination of NR protein levels in *cop1-4* and *35S-DN-COP1-Myc_6_* transgenic plants. Total proteins were extracted from wild-type, *cop1-4*, and *35S-DN-COP1-Myc_6_* transgenic plants grown for 15 d, and NR proteins (NIA1+NIA2) were detected by immunoblotting with an anti-NR antibody; tubulin was used as a loading control; (**C**) Cell-free degradation analysis of NR proteins in *cop1-4* and *cop1-6*. Total proteins were extracted from wild-type and *cop1-4* plants treated with 100 μM cycloheximide for the indicated times and NR proteins (NIA1 + NIA2) were detected by immunoblotting with an anti-NR antibody; tubulin was used as a loading control; (**D**) Yeast two-hybrid analysis of the interactions between COP1 and NR proteins. Full-length *COP1* was fused to sequences encoding the Gal4 activation domain (AD) in pGAD424. *NIA1* and *NIA2* cDNA were fused to sequences encoding the Gal4 DNA-binding domain (BD) in pGBT8. The constructs were transformed into yeast strain AH109. Numbers indicate yeast cells transformed with only pGAD424 and pGBT8, or with recombinant plasmids. Transformants were plated onto minimal medium, −Leu/−Trp and −Leu/−Trp/−His (5 mM 3-AT), and incubated for 4 d prior to observation.

Despite induction of *NR* expression by ammonium treatment (Table 1), NR protein levels were rather reduced (Figure 4), suggesting that NR protein levels are post-translationally regulated via modification with small or large molecules before degradation. In eukaryotes, various target proteins are destabilized by ubiquitination. Therefore, to examine whether NR proteins are destabilized by ubiquitination, we first investigated whether NR proteins could be degraded by the 26S proteasome complex. We examined NR protein levels in wild-type plants treated with the 26S proteasome inhibitor, MG-132. NR protein levels increased after MG-132 treatment (Figure 5A), indicating that these proteins are degraded by the 26S proteasome complex after polyubiquitination through E3 ubiquitin ligase activity. Therefore, we evaluated the effect of COP1 on NR protein levels. We chose COP1 because it has broad-spectrum E3 ubiquitin ligase activity for various proteins, including photoreceptors and transcription factors [38]. We examined the effect of COP1 on NR protein levels by immunoblotting using total proteins from the leaves of wild-type, *cop1-4*, and transgenic plants overexpressing COP1-Myc_6_ or DN-COP1-Myc_6_. *cop1-4* mutant contains a stop codon at amino acid position 283, which results in a loss of nuclear localization signal (NLS) and tryptophan–aspartic acid 40 (WD40) domain [42]. NR protein levels were much higher in *cop1-4* and DN-COP1-Myc_6_-overexpressing plants than in wild-type plants (Figure 5B). To investigate whether the stability of NR proteins is regulated by COP1 activity, we performed a cell-free degradation assay using wild-type and *cop1-4* plants. We evaluated the degradation rates of NR proteins in the presence of cycloheximide in wild-type versus *cop1-4* plants. The NR proteins degraded much more slowly in *cop1-4* than in wild-type plants (Figure 5C), indicating that NR proteins are destabilized by COP1. The higher levels of NR proteins in *cop1-4* and DN-COP1-Myc_6_-overexpressing plants suggest that NR protein levels are post-translationally regulated by COP1. Therefore, we examined possible interactions between COP1 and NR proteins by yeast two-hybrid analysis; however, COP1 did not interact with NIA1 or NIA2 (Figure 5D).

## 3. Discussion

Proper spatial arrangement of proteins is required for many cellular processes, including signaling, growth, and development. Proteins are distributed in specific subcellular compartments depending on their functions, and their subcellular localization is often mediated by interacting partners or determined by post-translational modifications [43]. Therefore, the spatiotemporal positioning of proteins is important for the precise control of their role and fate.

Prokaryotic NR proteins are classified into three distinct types: dissimilatory periplasmic nitrate reductases, respiratory membrane-bound nitrate reductases, and assimilatory cytoplasmic nitrate reductases [44]. Plant NRs localize to the cytoplasm and membrane [32,40,41]. Plant E3 SUMO ligases, including rice OsSIZ1 and OsSIZ2, and Arabidopsis AtSIZ1, localize to the nucleus, including nuclear bodies [16,24,45,46,47]. Most SUMO1- or SUMO2-conjugates are also found in the nucleus [33]. The Arabidopsis NR proteins, NIA1 and NIA2, interact directly with and are sumoylated by AtSIZ1 [17], suggesting that AtSIZ1 and NR proteins might have a similar cellular localization. Therefore, in the current study, we examined localization of AtSIZ1 and NR proteins by transient expression analysis using onion epidermal cells and fluorescently labeled fusion proteins. Interestingly, NR proteins NIA1 and NIA2 were detected in the cytoplasmic membrane when expressed individually (Figure 1A and Figure 2A), but their localization changed to the nucleus when co-expressed with AtSIZ1 (Figure 1B and Figure 2B). This observation strongly suggests that NR proteins are relocalized by AtSIZ1, although there is currently no direct evidence for this. Treatment of NR proteins with their substrate, nitrate, did not affect their localization, regardless of whether they were expressed separately or co-expressed with AtSIZ1 (Figure 2A,B). Notably, however, these fluorescent signals were significantly reduced in ammonium-treated cells separately expressing the NR proteins or co-expressing them with AtSIZ1. Fluorescence was not detected in ammonium-treated cells under our experimental conditions (Figure 3A,B). Ammonium is an end product of the nitrate reduction pathway, indicating that NR activity is not required if sufficient ammonium is present. Therefore, it is possible that NRs are degraded if ammonium is supplied exogenously. These results strongly suggest that NR protein levels are post-translationally regulated by nitrogen sources.

Thus, we examined the effect of nitrate and ammonium on the NR gene expression and protein levels. *NIA1* and *NIA2* expression was induced in nitrate- and ammonium-treated leaves and roots (Table 1). However, compared with the control, NR protein levels were high in nitrate-treated leaves and roots, but much lower in ammonium-treated leaves and roots (Figure 4). These results indicate that transcription of *NIA1* and *NIA2* is induced by nitrate, although it is currently unclear whether nitrate affects the stability of NR proteins at the post-translational stage. The increased *NIA1* and *NIA2* transcript levels and reduced NIA1 and NIA2 protein levels in ammonium-treated leaves and roots (Table 1 and Figure 4) suggest that the levels and stability of NR proteins are regulated via post-translational modification. Phosphorylation decreases NR activity [28,48] and sumoylation stimulates it [17], indicating that sumoylation and phosphorylation have antagonistic effects on NR activity. Moreover, AtSIZ1 stabilizes NR proteins [17]. These observations and the current results suggest that nitrate induces sumoylation of NR proteins, thereby increasing their stability and activity, and ammonium induces their phosphorylation, leading to degradation and inactivation. However, the effects of nitrate and ammonium on phosphorylation and sumoylation of NR proteins have not yet been experimentally confirmed.

Previous studies show that AtSIZ1 localizes to the nucleus under the conditions tested [16,24,39,40,41]. However, we found that its localization could change. Specifically, the subcellular localization of AtSIZ1 was altered by nitrate treatment, as we detected this protein not only in the nucleus, but also in the cytoplasm in the form of bodies (Figure 2B). Currently, we do not know why AtSIZ1 localizes to the cytoplasm as bodies after nitrate treatment. We also do not know whether the activity and stability of cytoplasm-localized AtSIZ1 differ from those of nucleus-localized AtSIZ1 or whether its cytoplasmic localization affects stability and function of NR proteins. We previously found that AtSIZ1 levels are not affected by nitrate [16], indicating that some portion of the AtSIZ1 pool moves from the nucleus to the cytoplasm. Therefore, examining the effect of the nitrate-specific movement of this protein from the nucleus to cytoplasm on the activity or stability of NR proteins would shed light on how the sumoylation system participates in nitrogen assimilation via nitrate reduction by NR proteins.

Our transient expression assay using onion epidermal cells and immunoblotting strongly suggested that NR protein levels are post-translationally controlled by ammonium. NR proteins indeed accumulated in *cop1-4* and DN-COP1 transgenic plants (Figure 5B), but no interaction between COP1 and NR proteins was observed (Figure 5D). However, NR protein levels increased after treatment with a 26S proteasome complex inhibitor (Figure 5A). Moreover, a cell-free degradation assay revealed that degradation of NR proteins occurred later in *cop1-4* than in wild-type (Figure 5C). These results strongly suggest that NR protein levels are post-translationally regulated by the ubiquitination/proteasome pathway, although their levels are not directly regulated by COP1. HY5 is a direct target of COP1 and is accumulated in *cop1-4*, *cop1-5*, and *cop1-6* mutants [38,49,50]. *NIA2* expression is positively regulated by HY5 and HYH [31]. NR transcript levels were higher in etiolated *cop1* seedlings and in green leaves of 3-week-old *cop1* plants than in wild-type [36]. These findings indicate that COP1 is involved in degradation of a positive regulator of NR expression because COP1 did not interact with NR proteins. All of these findings, including the current results, indicate that NR protein levels are high in *cop1-4* and DN-COP1 transgenic plants due to high HY5 levels in these plants; they also suggest that there must be other E3 ubiquitin ligase(s) for NR proteins and that NR levels are directly or indirectly post-translationally controlled by E3 ubiquitin ligases, including COP1. However, we cannot rule out the possibility that NR protein levels could be controlled by a COP1-containing E3 ubiquitin ligase complex, such as cullin-based ubiquitin ligase [51].

**Figure 6:**
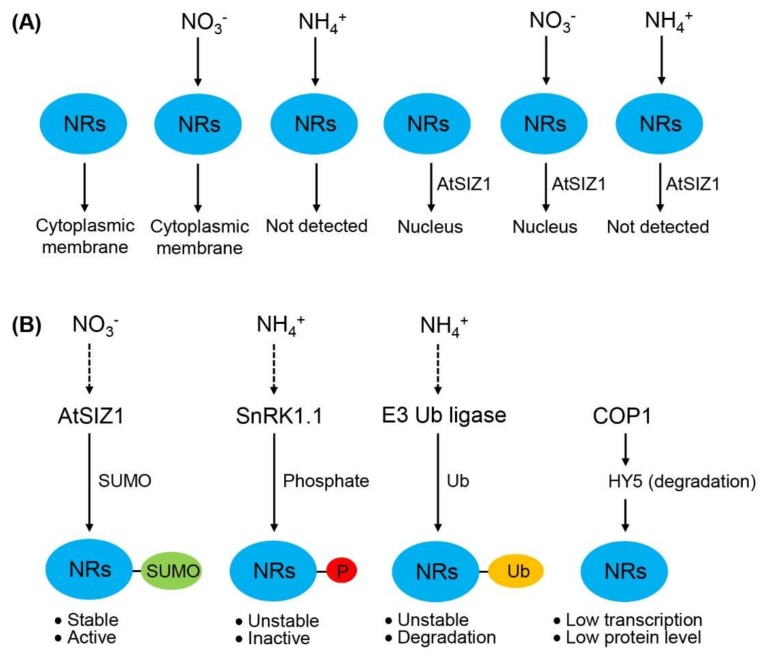
Schematic representation showing the localization, level, and activity of nitrate reductases (NRs). (**A**) Regulation of the localization of NR proteins by E3 SUMO ligase and nitrogen sources. NR proteins localize to the cytoplasmic membrane in untreated cells. This localization is unaffected by nitrate (NO_3_^−^) treatment. However, ammonium (NH_4_^+^) treatment degrades NR proteins; consequently, NR proteins are not detected in ammonium-treated cells. When co-expressed with AtSIZ1, NR proteins localize to the nucleus and are similarly affected by nitrate and ammonium treatment as in the absence of AtSIZ1; (**B**) Regulation of the level and activity of NR proteins by post-translational modifications. Sumoylation stimulates NR activity, whereas phosphorylation and ubiquitination reduce NR level and stability, leading to reduced NR activity. COP1 represses NR expression by destabilizing HY5, leading to reduced NR level. Dashed arrows indicate confirmed mechanisms, and dotted arrows indicate putative mechanisms. NRs, nitrate reductases; AtSIZ1, E3 SUMO ligase; SnRK1.1, SNF1-related kinase; COP1, E3 ubiquitin ligase; HY5, transcription factor; P, phosphate; Ub, ubiquitin.

Together, the results of previous studies and this study indicate that the localization of NR proteins is influenced by AtSIZ1 and ammonium (Figure 6A), and the level and activity of NR proteins are regulated by post-translational modifications, such as sumoylation, phosphorylation, and ubiquitination (Figure 6B). These data also suggest that nitrate stabilizes or activates NR proteins, whereas ammonium destabilizes or inactivates them (Figure 6B).

NR proteins also act on other substances. For example, NRs of *Escherichia coli*, *Rhodobacter sphaeroides*, *Ralstonia eutropha*, *Paracoccus denitrifications*, and *P. pantotrophus* use selenite and tellurite as electron acceptors, reducing them to selenate and tellurite, respectively [52,53,54]. Additionally, low levels of NO due to the *nia1nia2* mutation enhance selenite sensitivity of Arabidopsis, suggesting that NR activity is directly or indirectly responsible for selenite tolerance [55]. Additionally, it has been recently reported that nitrate inhibits selenium reduction because of its high reduction potential and/or low selenite/selenate reductase activity, indicating that nitrate reduction affects selenium reduction [56]. These data suggest that plant NR proteins can also reduce other substances, including selenate and tellurite, as well as nitrate selenate and tellurite, as well as nitrate, and the level and localization of NR proteins can also be influenced by them.

In conclusion, AtSIZ1 is responsible for the nuclear localization of NR proteins, and ammonium negatively regulates their levels. In addition, NR proteins are degraded by the ubiquitination system, and COP1 indirectly induces NR degradation. Experiments involving the isolation of an E3 ubiquitin ligase for NR proteins should help elucidate how their levels are regulated by specific nitrogen sources after translation. Further studies on the effect of nitrate on AtSIZ1 localization and the effect of COP1 on NR levels should help uncover how AtSIZ1 modulates nitrogen assimilation by stimulating NR activity and how ubiquitination pathways, including pathways involving COP1 and other E3 ubiquitin ligases, control NR-mediated nitrogen assimilation by inducing their degradation.

## 4. Materials and Methods

### 4.1. Plant Material and Culture Conditions

*Arabidopsis thaliana* ecotype Columbia-0 (Col-0; wild-type), *cop1-4*, and dominant negative (DN)-COP1 transgenic plants were used in this study. The transgenic plants were produced by introduction of DN-COP1 into the Arabidopsis ecotype Columbia-0. The plants were grown in a growth chambers at 22 °C under 16 h light/8 dark cycle on 0.75% agar medium containing Murashige and Skoog (MS) salts, 0.5 g L^−1^ MES, and 10 g L^−1^ sucrose. The experiments were repeated three times, unless otherwise mentioned.

### 4.2. Subcellular Localization

The *CFP* (cyan fluorescence protein) coding sequence was fused in-frame to the 5′-ends of *NIA1* and *NIA2*, and the *YFP* (Yellow fluorescence protein) coding sequence was fused in-frame to the 5′-end of AtSIZ1. Fusion genes encoding CFP and YFP were expressed under the control of the 35S promoter. Three micrograms of each plasmid were transferred into the epidermis on the inner surfaces of onion scales via particle gun-mediated bombardment (Bio-Rad, Hercules, CA, USA). The bombarded tissues were incubated at 25 °C in the dark for 16 h in a solution containing 5 mM KNO_3_ or 5 mM (NH_4_)_2_SO_4_. CFP and YFP fluorescence were observed by confocal laser scanning microscopy.

### 4.3. Quantitative RT-PCR

The expression levels of *NIA1* and *NIA2* were examined by quantitative RT-PCR. PCR was performed using 2 µg of total RNA from the shoots or roots of wild-type plants treated with or without nitrogen sources, as previously described [17]. The primers used for PCR were as follows: *NIA1*, 5′-GCTAGTAAGCATAAGGAGAGGCTA-3′ and 5′-CCTTCACGTTGTAACCCATCTTC-3′; *NIA2*, 5′-TGTCTCAGTACCTAGACTCTTTGC-3′ and 5′-GACGTACATTTCAGTCTCATCCTC-3′. All reactions were repeated three times with three independent RNA samples.

### 4.4. Examination of NR Protein Levels

To investigate the effects of different nitrogen sources on NR protein levels, wild-type seeds were germinated and grown on MS agar medium. After 15 d, the samples were incubated in a solution containing 5 mM K_2_SO_4_, KNO_3_, or (NH_4_)_2_SO_4_ for 15 h. After total proteins were extracted from the leaves of each sample, the proteins were separated by 8% SDS-PAGE and expression of NR proteins NIA1 and NIA2 was examined by immunoblotting with a cucumber anti-NR antibody (0.5 μg/mL) (antibodies-online), as previously described [17]. 

To examine the effect of a 26 proteasome complex inhibitor on NR levels, wild-type plants grown on MS medium for 15 day were treated for 1 h with 100 μM MG132 (*N*-(benzyloxycarbonyl)-Leu-Leu-Leu-al) (Sigma-Aldrich, St. Louis, MO, USA). Total proteins were extracted from each sample, separated in 8% SDS-polyacrylamide gels, and NR proteins detected by immunoblotting with anti-NR antibody. 

*Cop1-4* and DN-COP1-overexpressing plants were used to examine the effect of COP1 on NR levels. The *cop1-4* mutant and *35S-DN-COP1-Myc_6_* transgenic plants were kindly provided by Dr. Nam-Hai Chua (Rockefeller University, USA). Total proteins were extracted from the leaves of wild-type, *cop1-4*, and *35S-DN-COP1-Myc_6_* transgenic plants grown for 15 d on MS medium, and NR protein levels were examined as described in above.

### 4.5. Cell-Free Degradation Assay

To examine the effect of COP1 on stability of NR proteins, wild-type and *cop1-4* plants grown on MS medium for 15 d were treated with 100 μM cycloheximide and samples were collected at the indicated time points. Total proteins were extracted from each sample, separated in 8% SDS-PAGE gels, and NR proteins detected by immunoblotting with an anti-NR antibody.

### 4.6. Yeast Two-Hybrid Experiments

Yeast two-hybrid assays were performed using the GAL4-based two-hybrid system (Clontech, Mountain View, CA, USA). Full-length *COP1, NIA1*, and *NIA2* cDNA sequences were cloned into pGAD424 containing an activation domain (AD) and pGBT8 containing a binding domain (BD) (Clontech) to generate the constructs *AD-COP1*, *BD-NIA1*, and *BD-NIA2.* All constructs were transformed into yeast strain AH109 using the lithium acetate method and the cells were grown on minimal medium (−Leu/−Trp). The transformants were plated on minimal medium (−Leu/−Trp/−His/5 mM 3-AT (3-amino-1,2,4-triazole)) to test the interactions between COP1 and NIA1 or NIA2.

## Figures and Tables

**Table 1:** Relative transcript levels of *NIA1* and *NIA2* in the leaves and roots of WT plants supplied with specific nitrogen source.

	Leaf					Root			
	None	K_2_SO_4_	KNO_3_	(NH_4_)_2_SO_4_	None	K_2_SO_4_	KNO_3_	(NH_4_)_2_SO_4_
*NIA1*	1.00 ± 0.18	1.02 ± 0.15	3.33 ± 0.27	2.23 ± 0.24	1.00 ± 0.12	1.10 ± 0.21	1.62 ± 0.44	1.56 ± 0.07
*NIA2*	1.00 ± 0.19	1.01 ± 0.13	2.00 ± 0.11	1.36 ± 0.12	1.00 ± 0.13	0.98 ± 0.14	1.59 ± 0.36	1.34 ± 0.08
